# Surface Characterization of New β Ti-25Ta-Zr-Nb Alloys Modified by Micro-Arc Oxidation

**DOI:** 10.3390/ma16062352

**Published:** 2023-03-15

**Authors:** Pedro Akira Bazaglia Kuroda, Carlos Roberto Grandini, Conrado Ramos Moreira Afonso

**Affiliations:** 1 Materials Engineering Department (DEMa), Universidade Federal de São Carlos (UFSCar), São Carlos 13565-905, SP, Brazil; 2 Laboratório de Anelasticidade e Biomateriais, UNESP—Universidade Estadual Paulista, Bauru 17033-360, SP, Brazil

**Keywords:** micro-arc oxidation, surface modifications, titanium alloys

## Abstract

The technique of surface modification using electrolytic oxidation, called micro-arc oxidation (MAO), has been used in altering the surface properties of titanium alloys for biomedical purposes, enhancing their characteristics as an implant (biocompatibility, corrosion, and wear resistance). The layer formed by the micro-arc oxidation process induces the formation of ceramic oxides, which can improve the corrosion resistance of titanium alloys from the elements in the substrate, enabling the incorporation of bioactive components such as calcium, phosphorus, and magnesium. This study aims to modify the surfaces of Ti-25Ta-10Zr-15Nb (TTZN1) and Ti-25Ta-20Zr-30Nb (TTZN2) alloys via micro-arc oxidation incorporating Ca, P, and Mg elements. The chemical composition results indicated that the MAO treatment was effective in incorporating the elements Ca (9.5 ± 0.4 %atm), P (5.7 ± 0.1 %atm), and Mg (1.1 ± 0.1 %atm), as well as the oxidized layer formed by micropores that increases the surface roughness (1160 nm for the MAO layer of TTZN1, 585 nm for the substrate of TTZN1, 1428 nm for the MAO layer of TTZN2, and 661 nm for the substrate of TTZN2). Regarding the phases formed, the films are amorphous, with low crystallinity (4 and 25% for TTZN2 and TTZN1, respectively). Small amounts of anatase, zirconia, and calcium carbonate were detected in the Ti-25Ta-10Zr-15Nb alloy.

## 1. Introduction

In the biomedical field, metallic implants to replace and repair rigid tissues, such as cortical bone, titanium, and its alloys, are highlighted as raw materials in manufacturing new implants [1,2]. It is known that titanium has a suitable density/mechanical ratio property, a low elastic modulus value reducing the stress shielding effect, good biocompatibility, and good corrosion resistance in body fluids due to a small passivation layer of titanium dioxide (TiO_2_) [3,4]. Optimizing the characteristics of titanium and its alloys as a biomaterial is extremely important to increase implant life [5]. One of the alternatives is to functionalize the surface of the implants by incorporating essential elements for bone growth, such as calcium and phosphorus, using the technique known as micro-arc oxidation (MAO) or also known as plasma electrolytic oxidation (PEO) [6,7,8].

MAO is a technique that makes it possible to obtain a porous disordered surface. Several variables and parameters can influence film growth, such as the applied potential difference between the electrodes [9], the maximum current established, surface treatment time, and the chemical composition of the electrolyte [10,11,12]. A concentrated electrolyte causes the surface to incorporate a more significant number of elements; increasing the treatment voltage can modify the morphology, film thickness, crystalline structure, and chemical composition (the higher the voltage, the greater the intensity of the electric arc created, therefore, the greater its ability to fuse elements on the implant surface). In addition, increasing the tension can cause the films to crack due to the increase in temperature and pressure during the MAO process [9].

Other surface modification techniques, such as sputtering, anodizing, and thermal oxidation, are also used in titanium alloys to functionalize the surface. However, the most significant advantage of using the micro-arc technique is that the film formed is dense, thick, adherent, has low contamination, and enables the incorporation of essential elements, particles, and compounds for the adhesion and growth of bone tissue, unlike the anodizing technique that produces films formed by nanotubes with low adherence, making it challenging to handle the sample, the sputtering technique which has high contamination and hardens the surface, and thermal oxidation which, even though it is also a simple technique, does not allow the incorporation of particles in the produced oxide film.

Thus, this work aimed to fabricate a ceramic surface of Ti-25Ta-10Zr-15Nb (TTZN1) and Ti-25Ta-20Zr-30Nb (TTZN2) alloys using electrochemical treatment by MAO. The TTZN1 and TTZN2 alloys are substitutes for TNZT (Ti-29Nb-13Ta-4.6Zr), recalling that TNZT has β-phase predominance and elastic modulus values of approximately 65 GPa [13,14], a value very close to that of a human cortical bone, 30 GPa.

The TTZN1 and TTZN2 alloys are unprecedented in the current literature. The scientific community still needs to investigate their physical, mechanical, and chemical characteristics. However, other titanium alloys containing tantalum, zirconium, and niobium, with different mass percentages from those proposed in this work, have already been developed and studied in the scientific community [15]. The concentration of each element is chosen to stabilize the β phase, as tantalum and niobium are considered β-stabilizing elements. Generally, β-type titanium alloys tend to have a low modulus of elasticity, avoiding the “stress shielding effect” [16]. Among the TNZT system alloys, two beta alloys stand out: Ti-35.3Nb-5.1Ta-7.1Zr, which has an elastic modulus value of 55 GPa, and Ti-29Nb-13Ta-4.6Zr (aged) has a high elastic modulus value for 80 GPa but an excellent tensile strength value (911 MPa) [17].

## 2. Materials and Methods

For the melting of Ti-25Ta-10Zr-15Nb (TTZN1) and Ti-25Ta-20Zr-30Nb (TTZN2) alloys, an arc furnace was used with a water-cooled copper crucible, a nonconsumable tungsten electrode, and a controlled argon atmosphere [18]. Due to the high melting point of the elements, melting was performed 10× to ensure good homogeneity.

To carry out the surface modification process via MAO, an aqueous solution comprised calcium acetate monohydrate (C_4_H_6_CaO_4_ H_2_0) of Reagen/Ultrapure Chemical of Brazil, β-glycerol phosphate (C_3_H_7_Na_2_O_6_P 5H_2_0) from Sigma-Aldrich (St. Louis, MO, USA) and magnesium acetate tetrahydrate (C_4_H_6_MgO_4_ 4H_2_O) from Sigma-Aldrich at a molar concentration of 0.35, 0.02, and 0.1 M, respectively [19]. The electrolyte was stirred for approximately four hours to ensure a good homogeneity of the solution. The MAO conditions consisted of a Keysight continuous voltage source, model N5751A (Oulu, Finland), applying a potential of 300 V and a current limited to 2.5 A, duration of 1 min, and platinum foil (Pl) as a cathode. To record the current, an Agilent benchtop digital multimeter, flexible cables, and a computer were used to make the recordings. Treatment was carried out in potentiostatic regimes.

After the treatments, the alloys were characterized by X-ray diffraction (XRD) measurements using a MiniFlex600 diffractometer (Rigaku, Tokyo, Japan) with Cu Kα radiation (λ = 1.54056 Å). The data were collected using the powder method and the fixed-time mode, with steps of 0.04°, ranging from 20 to 100°, 2θ step sizes, and a 10°/min collection time. To obtain the scanning electron microscopy images, a Carl Zeiss electron microscope (Oberkochen, Germany), model EVO-015, was used, and topography and roughness (Ra) were measured using confocal laser scanning microscopy, CLSM (Leica, DC3M, Wetzlar, Germany).

The XPS technique analyzed chemical analyses of the surfaces with X-ray excited photoelectron spectroscopy, using a Thermo Scientific Inc. AlKa (Waltham, MA, USA), at National Nanotechnology Laboratory (LNNano—Brazil).

Wettability and surface energy were obtained using contact angle measurements using the droplet technique on a Krüss DSHAT HTM Reetz GmbH goniometer at Department of Materials Engineering of the Federal University of São Carlos (UFSCar), São Carlos, Brazil. The experiments were carried out under environmental conditions, taking the average value of three measurements at different locations of the samples. Deionized water (polar liquid) and glycerol (nonpolar liquid), 2.5 μL volume, were used as the test fluid. The surface energy was calculated according to the Owens–Wendt method [20].

## 3. Results and Discussion

**Figure 1:**
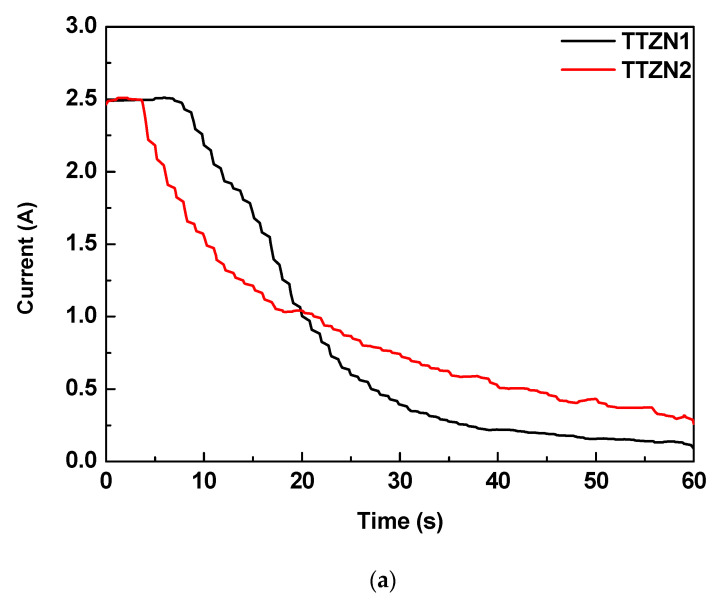

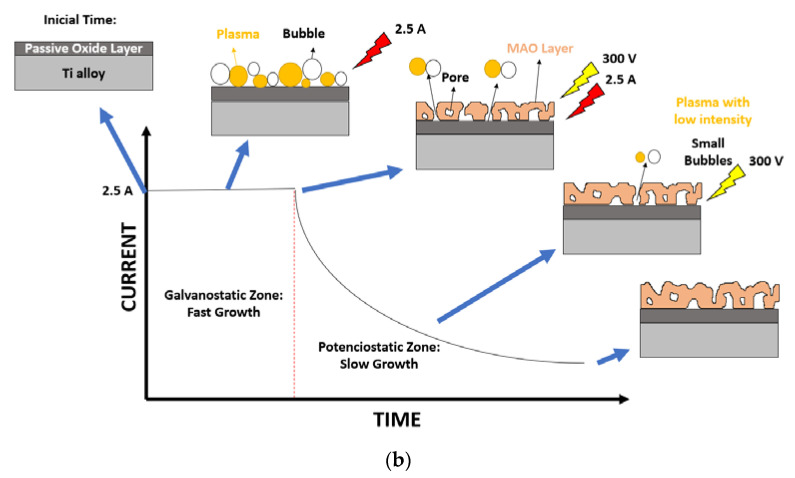
Current variation during the MAO process on TTZN1 and TTZN2 alloys (**a**) and film growth mechanism via MAO treatment (**b**).

Figure 1 shows a current versus time curve in the MAO process performed on the β TTZN1 and TTZN2 alloys. To carry out this surface modification process, the electric current of 2.5 A was set as a limit. In the initial stages, the current remains at 2.5 A as the oxide layer is still growing and does not have sufficient electrical resistance to reduce the current value. At a given moment, there is a decrease in the current value due to the increase in the dielectric barrier. In the initial stages, the electric arcs have high energies with a high capacity to change the surface of the samples and incorporate the elements in the electrolyte. On the other hand, when there is an increase in the dielectric barrier of the oxide layer, there is a decrease in the current. Consequently, there is a reduction in the number of micro-arcs on the surface, reducing the surface alteration capacity [8].

It can be observed that the β TTZN1 and TTZN2 alloys presented different curves. TTZN1 remains in the galvanostatic regime for longer than TTNZ2, which may indicate that the formed TTZN1 alloy layer has a higher electrical conductivity in the early stages. In addition, after the MAO surface modification process, it can be observed that the current of the TTZN2 alloy is still higher than that of the TTNZ1 alloy, and the decay of the current during the process is smoothed [8].

Analyzing the current versus time curve, it is observed that the TTZN2 alloy has a greater electrical charge involved in the system (area of the curve) during the MAO surface modification process.

**Figure 2:**
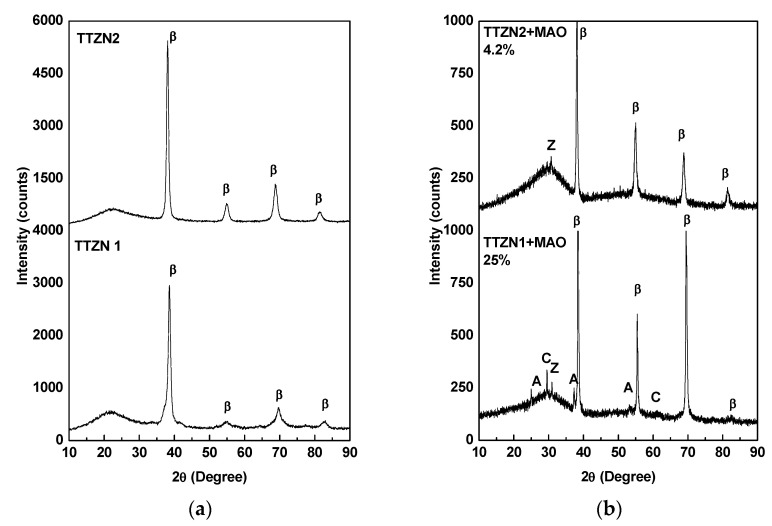
XRD pattern of β TTZN1 and TTZN2 after casting (**a**) and MAO treatment (**b**).

To obtain surface information, XRD patternswere obtained for the TTZN1 and TTZN2 alloys (Figure 2). After melting, the X-ray patterns show characteristic peaks of the body-centered cubic phase, β, in both alloys produced (Figure 2a). In titanium alloys, it is known that the β phase has a lower elastic modulus value, with a more significant potential to be used as an orthopedic biomaterial compared to α-type titanium alloys with a compact hexagonal crystalline structure. β-type TTZN1 and TTZN2 alloys are stable β-type due to their β-stabilizing fraction of niobium and tantalum [21]. For TTZN1, after the MAO technique (Figure 2b), showed the presence of the TiO_2_ anatase phase (A), calcium carbonate (C) in the trigonal form (CaCO_3_—a compound considered important for the formation of the hydroxyapatite), ZrO_2_ (Z) in the cubic allotropic form and the β phase of bulk substrate, as well as an amorphous halo can be observed. Studies in the literature report that the anatase phase of titanium dioxide can decrease bacterial colonization and induce the growth of hydroxyapatite on the surface of implants [7,22].

Sousa et al. developed alloys of the Ti-15Zr-Mo system (Mo = 0, 5, 10, and 15 wt%) and performed surface modification via MAO (300 V and 2.5 A) to functionalize the surface of the alloys with the elements calcium, phosphorus, and magnesium [19]. The XRD analysis showed that the formed ceramic layers have low crystallinity, and the crystalline phases are TiO_2_ anatase and rutile, tetragonal zirconia, and CaCO_3_. The detected phases are like those formed on the surface of TTZN1 and TTZN2 alloys.

For the TTZN2 alloy, after MAO treatment, characteristic peaks of the bulk β Ti bcc phase and a peak of cubic zirconium oxide can be observed. Note that from the XRD results, the MAO surface of TTZN2 is amorphous, with 4.2% crystallinity, which may result from the high temperatures and pressure reached during the production of electric arcs [4]. The TTZN1 alloy showed 25% crystallinity on the surface coating.

In all XRD patterns, it is possible to observe an amorphous halo, which could be a result of the high temperature and pressure on the surface during the plasma sparkling [23], and knowing the crystallinity of the surface formed is interesting as crystalline and amorphous materials have a different atomic organization, as a consequence the energy involved in the structures formed are different, influencing the surface energy and contact angle.

**Figure 3:**
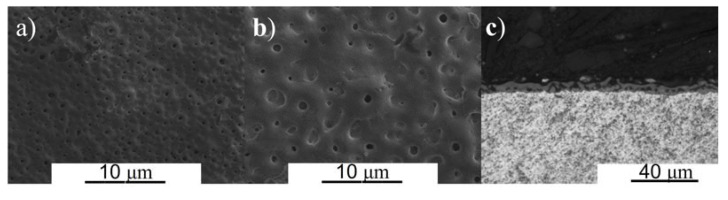
Micrographs of the TTZN1 alloy surface obtained using SEM posterior MAO treatment with different magnifications: 1000× (**a**), 3000× (**b**), and cross-sectional image (**c**).

**Figure 4:**
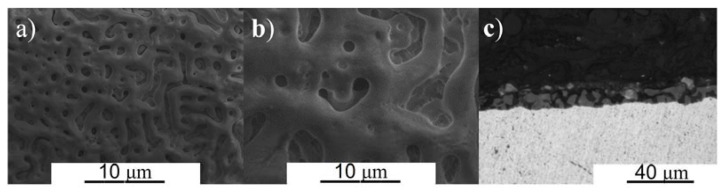
Micrographs of the TTZN2 alloy surface obtained by SEM posterior MAO treatment with different magnifications: 1000× (**a**), 3000× (**b**), and image of cross-section (**c**).

The morphology and cross-sectional SEM images are shown in Figure 3 and Figure 4 for β TTZN1 and TTZN2 alloys, respectively. The formation of tiny pores in the films can be observed that are formed via MAO and different thicknesses as a function of the concentration of alloying elements. The TTZN1 alloy has smaller pores with circular shapes, while TTZN2 has circular shapes and some lamellae. Some studies report that the porous surfaces formed by MAO improve wear resistance [24], corrosion [25], the bioactivity of implants [26], and osseointegration capability to the substrate.

Regarding the cross-sectional images, the micrographs inform that the addition of niobium and zirconium in the alloys increased the size of the layer of the formed film, and the film formed on the surface of the TTZN1 alloy is more compact (without many pores and bubbles). The TTZN1 alloy has a film thickness of approximately 6.8 ± 0.1 μm, and the TTZN2 alloy has a thickness of 12.7 ± 2.7 μm.

Kaseem and Choe produced MAO-treatment Ti-xNb alloys [27]. In their studies, the micropores’ average size and distribution showed significant variation with the Nb content. For alloys with Nb content above 30% by weight, large craters with structures and cracks similar to the images observed in Figure 4 were observed. The authors infer that changes in the coating structure are mainly attributed to variations in the size and intensity of the plasma discharges associated with the Nb content. The greater the amount of Nb in the alloy, the greater the ability for pore fusion due to the high energies of the electric arcs.

**Figure 5:**
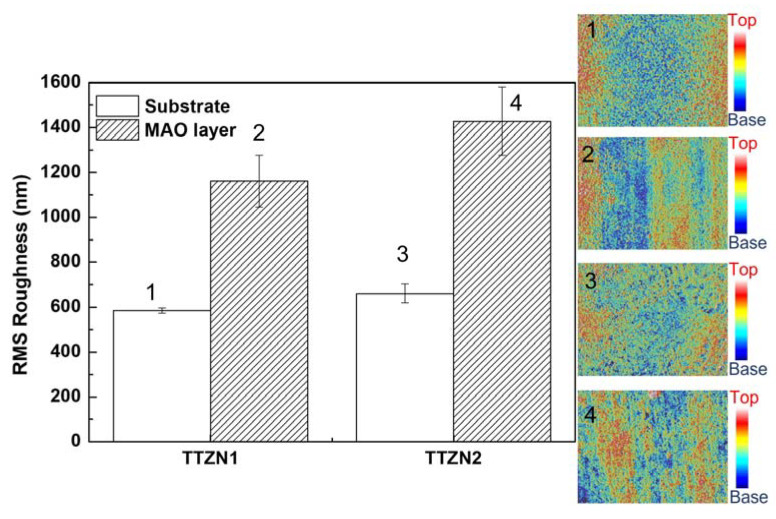
Comparative results of the roughness of TTZN1 and TTZN2 surfaces before and after MAO treatment obtained through confocal laser scanning microscopy.

The confocal laser scanning microscopy, CLSM analysis performed in three distinct regions, allowed the roughness measurements of the films and the substrate, and the results are shown in Figure 5. As expected, the surface modification increased the surface roughness, and the addition of zirconium and niobium in the alloys also increased the roughness, corroborating the scanning electron microscopy images. The alloy with the highest amount of zirconium and niobium (TTZN2) has two morphologies, pores and gaps, increasing the surface roughness. Recent studies have shown that adding Nb from the Ti–Nb and Ta system alloys to the Ti–Ta system increases the surface roughness modified via MAO [27,28].

**Figure 6:**
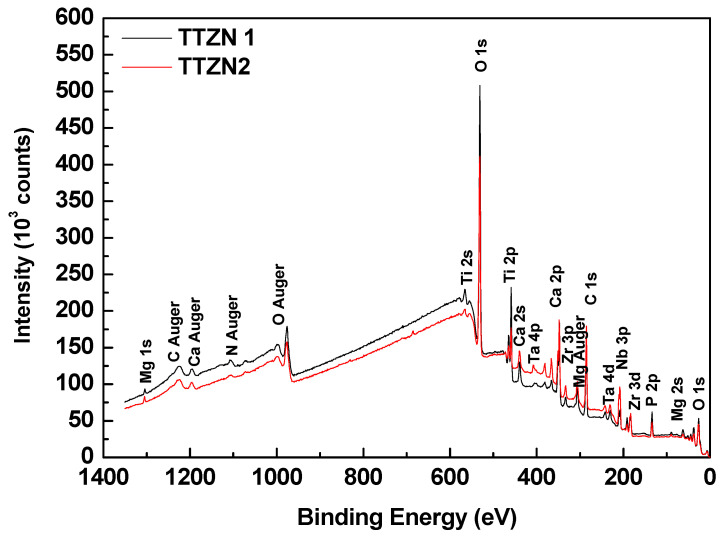
Comparison between survey spectra obtained using XPS for TTZN1 and TTZN2 after MAO treatment.

**Figure 7:**
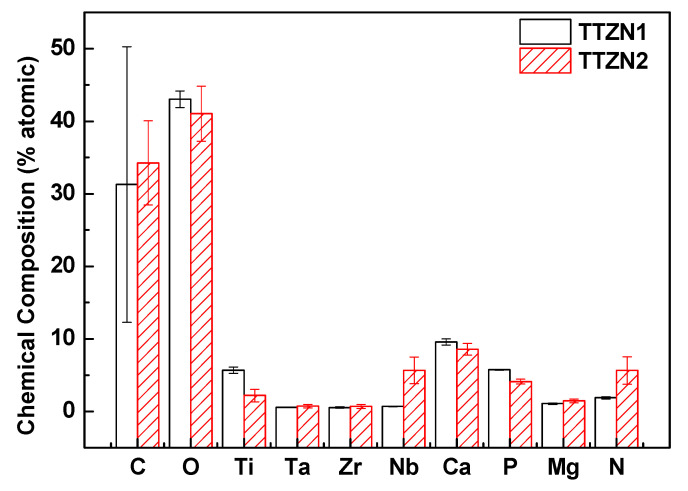
Elemental quantification obtained using XPS for TTZN1 and TTZN2 alloys.

To better understand the characteristics of the films, XPS measurements were performed in three different regions in each sample. Figure 6 shows a typical spectrum of an XPS survey mode. The images show that the detected elements consisted mainly of Ti, Ta, Nb, Zr, O, and C. Traces of the elements present in the electrolyte (Ca, P, and Mg) were also observed. Figure 7 shows the atomic percentage of the elements detected using the technique on the measured surfaces.

The chemical composition of the surface via XPS was performed to identify whether the elements in the electrolyte are present in the films (calcium, phosphorus, and magnesium). The results showed that in addition to the presence of the alloy precursor metals (titanium, tantalum, zirconium, and niobium), there is also the presence of the electrolyte elements, indicating that the electric arcs produced in the MAO process were able to melt the elements, showing that the process was carried out efficiently when incorporating elements that could lead to a better biological response from the host. Analyzing the values in the table, it can be observed that there was a decrease in the amount of Ti, Ca, and P with the addition of Nb and Z in the alloys. The element carbon detected by the XPS comes from the electrolyte solution, as magnesium acetate, calcium acetate, and β-glycerol phosphate have carbon in their chemical composition.

**Figure 8:**
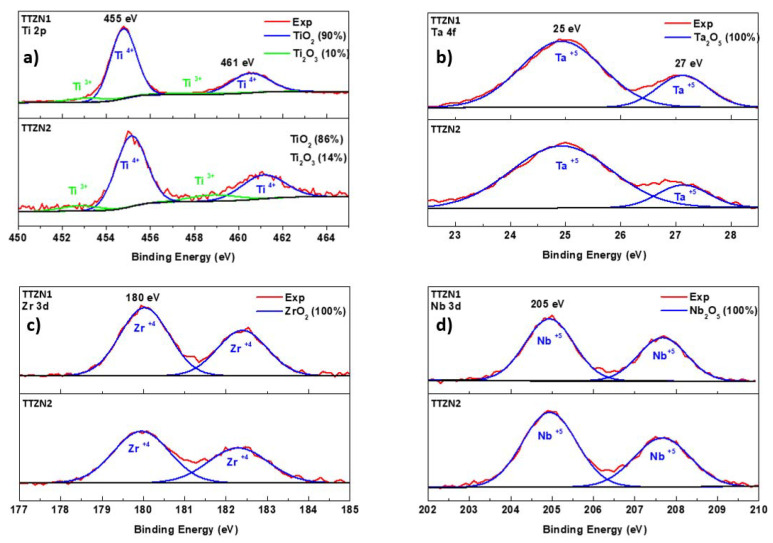
High-resolution spectrum of Ti 2p (**a**), Ta 4f (**b**), Zr 3d (**c**), and Nb 3d (**d**) for TTZN1 and TTZN2 alloys.

Figure 8 shows the high-resolution XPS spectra performed on the β TTZN1 and TTZN2 alloys. The high-resolution spectra of Ti 2p (Figure 8) are typically shown to be composed of a doublet 2p3/2 and 2p1/2. Ti 2p peaks are present in all the analyses performed on the alloys, indicating that the titanium oxide present on the alloy surface is mainly in the form of TiO_2_ [29,30]. The high-resolution spectra of tantalum (Figure 8b) show doublets composed of 4f7/2 and 4f5/2, representing the energies related to Ta_2_O_5_ oxide [31]. The niobium spectra (Figure 8c) showed peaks of Nb 3d, 3d5/2, and 3d3/2 doublets in the Nb+5 oxidizing state of Nb_2_O_5_. For the analysis of zirconium (Figure 8d), the spectra show doublets comprising two components, Zr 3d5/2 and Zr 3d3/2, of the ZrO_2_ formed by the reaction between the metal ions of Zr4+ and the oxygen ions O_2_ [32,33].

**Figure 9:**
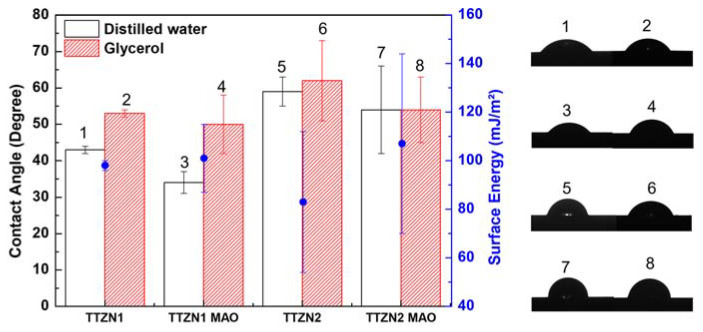
Comparative contact angle and surface energy values for TTZN1 and TTZN2 alloys.

Figure 9 shows the results related to the contact angle and surface energy tests. All samples analyzed in the condition without surface modification showed hydrophilic behavior (<90°), and hydrophilic surfaces tend to improve the initial stages of cell adhesion, proliferation, differentiation, and bone mineralization compared to hydrophobic surfaces [34]. The MAO surface modification process did not significantly change the contact angle values when compared with the bulk β TTZN1 and TTZN2 alloys.

The contact angle measured for the TTZN1 and TTZN2 alloys, after MAO, calculated using distilled water, showed an increased value for the TTZN2 alloy. The Nb and Zr additions to the alloy increased the surface’s crystallinity, the pores size, and the contact angle in the polar liquid. Chu, Li, and Liu (2020) explain that in MAO coatings with smaller pores and roughness and lower OH content, distilled water cannot wet the smaller pores on the surface of the MAO coating; that is, such morphology and surface tension of the water that leads to the capture of ambient gas at the interface between the surface of the MAO coating and the water used for the contact angle test [35]. The MAO surface modification process did not significantly change the contact angle’s values, which may indicate a similar rate of the initial stages of cell adhesion, proliferation, differentiation, and bone mineralization. However, the presence of calcium, phosphorus, and magnesium ions on the modified surfaces can improve the long-term biological response as they are fundamental elements in the formation of bone tissue.

## 4. Conclusions

From the results presented for the β TTZN1 and TTZN2 alloys, it can be concluded that the MAO coatings oxide films showed a porous structure with different pore sizes, and the addition of the alloying elements zirconium and niobium in the alloy promoted the increasing of roughness. The cross-sectional image of MAO coatings showed that the TTZN2 alloy with a higher film thickness than TTZN1 alloy. The MAO oxide films presented TiO_2_ (anatase and rutile), zirconia ZrO_2_, Ta_2_O_5_, and Nb_2_0_5_ phases arising from the oxidation of the substrate. In addition, the elements calcium, phosphorus, and magnesium were incorporated into the MAO coating structure through electric arcs. All MAO coating of β TTZN1 and TTZN2 alloys samples analyzed in different condition showed hydrophilic behavior with and without surface modification. As an indication for future work, analyzing the corrosion resistance, tribocorrosion, wear tests, and mechanical properties contributed to classifying the β TTZN1 and TTZN2 alloys for applications as biomaterials.

## Data Availability

The data presented in this study are available on request from the corresponding author.
